# A comparison of breast density measures between mothers and adolescent daughters

**DOI:** 10.1186/1471-2407-11-330

**Published:** 2011-08-02

**Authors:** Gertraud Maskarinec, Yukiko Morimoto, Yihe Daida, John Shepherd, Rachel Novotny

**Affiliations:** 1 Epidemiology Program, University of Hawaii Cancer Center, Honolulu, USA; 2 Department of Human Nutrition, Food and Animal Sciences, University of Hawaii at Manoa, Honolulu, USA; 3 Kaiser Permanente Center for Health Research, Honolulu, USA; 4 Department of Radiology and Biomedical Imaging, University of California-San Francisco, San Francisco, USA

**Keywords:** Breast density, breast development, hereditary factors, % total body fat, breast imaging

## Abstract

**Background:**

Based on the importance of breast density as a predictor of breast cancer risk, we examined the heritable component of breast measures in mothers and daughters using Dual Energy X-ray Absorptiometry (DXA).

**Methods:**

We recruited 101 mothers ≥30 years and their daughters aged 10-16 years through Kaiser Permanente Hawaii. Scans of both breasts were taken using a DXA system in research mode, calibrated to distinguish fibroglandular and fatty breast tissue. We calculated correlation coefficients between mothers and daughters for breast volume, absolute fibroglandular volume (FGV), and %FGV and performed multiple linear regression to include relevant covariates.

**Results:**

Breast volume and absolute FGV in daughters were lower than in mothers and were positively associated with % total body fat and Tanner breast stage. In contrast, %FGV in daughters was higher than in mothers and was inversely associated with % total body fat. Although unadjusted correlations between mothers and daughters were significant for breast volume and absolute FGV (r = 0.28 and *p *< 0.01 for both), models adjusted for demographic variables, Tanner stage, and % total body fat indicated significant associations only among the more mature girls (Tanner stages 4&5). There was no significant association between %FGV of mothers and daughters.

**Conclusions:**

These results indicate that the heritability of breast volume and amount of dense tissue is measurable in adolescence, but percent breast density shows no relation between mothers and daughters at that time. Further study of breast tissue composition during adolescence and in young women may enhance understanding of breast cancer risk later in life.

## Background

Mammographic density, the distribution of fat, connective, and epithelial tissue in the female breast, has been used as a biomarker for breast cancer risk because a high percentage of density on mammographic images was shown to confer a 4-6 fold higher risk for breast cancer [1]. Furthermore, it has been argued that the absolute amount of dense tissue is the true determinant of breast cancer risk [2,3]. Ethnic differences in breast density have been described; in particular, women of Asian ancestry have higher breast density than Caucasians due to their smaller breast size [4,5]. In addition to hormonal and anthropometric determinants, the proportion of the breast occupied by density, at a given age, is highly heritable [6]. Having family members with a history of breast cancer is associated with breast higher density [7,8]. Also, breast density was significantly correlated between sisters (r = 0.16-0.27) but not mother-daughter pairs (r = 0.02-0.11) in a cohort of families affected with breast cancer [9]. In a more recent study using magnetic resonance imaging (MRI), a significant correlation was observed between mothers and daughters [10]. In a twin study, the correlation for percent density was 0.63 for monozygotic pairs but only 0.27 for dizygotic pairs [11]. Associations of breast density with variant alleles in genes related to breast cancer risk have also been described [12]. Given the radiation exposure, screening mammograms are advised for women above 40 or 50 years of age depending on the recommending authority, risk status, and country and cannot be used to monitor breast composition in young women and girls, as the risk outweighs potential benefits in that age group [13]. Therefore, Dual X-ray absorptiometry (DXA) has been explored as a research tool with the advantage that it is commonly available, it is the reference standard for measuring whole-body tissue composition, it does not entail breast compression, and it uses an X-ray dose 10 times lower than mammography [14,15]. DXA models breasts as containing two volumes of tissue, fibroglandular volume (FGV) and fat. This differs from mammographic density, which is defined as the ratio of radiodense area to total area in the mammogram. We demonstrated in a pilot study of pubertal girls that all stages of breast development, as described by Tanner, can be imaged using DXA breast scans [16]. In a cross-sectional investigation among adult women, the correlation between mammographic and DXA density among adult women was 0.76 (*p  *< 0.0001) [17]. The present analysis examines the hypothesis that, due to its heritable component, breast density from DXA scans is correlated between mothers and adolescent daughters. To test the hypothesis, we analyzed the relation between breast density measured by DXA in 101 mothers and their 113 daughters representing the major ethnic groups in Hawaii.

## Methods

### Study design and procedure

**Figure 1:**
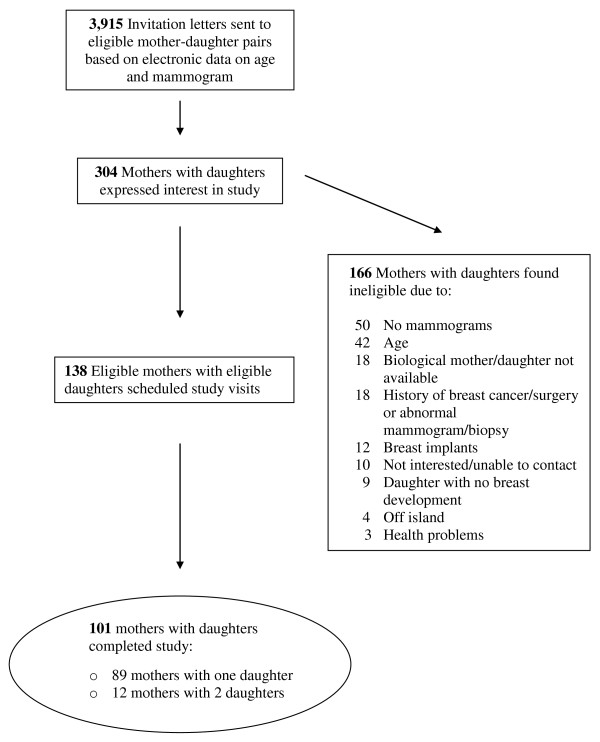
**Recruitment and study population**.

The current analysis was conducted as part of a mother-daughter study that measured breast density and body-fat composition in adult women and their adolescent girls using DXA [17]. The project was approved by the Committee on Human Studies at the University of Hawaii and the Institutional Review Board of Kaiser Permanente (KP) Hawaii. We recruited women aged 30 years and older through KP Hawaii (a large health maintenance organization) who had received a normal mammogram (BIRADS mammographic assessment categories 1 through 3) ordered by their health care provider in the last 2 years and their daughters aged 10-16 years. We mailed 3,915 invitation letters over 11 months (Figure 1). Potential participants were selected from the KP Hawaii membership data base according to the age and mammographic criteria. From the 304 respondents, we excluded mothers who had no mammogram, previous history of breast cancer or surgery, abnormal mammogram, previous biopsy, breast implants, or chronic health condition that interfered with study participation. We excluded girls without breast development as well as mother-daughter pairs who were not biologically-related or did not reside on the island of Oahu (where the DXA machine is located).

Of the 138 eligible mother-daughter pairs, 101 pairs, plus 12 additional daughters, completed the study. Prior to DXA scans, all participants signed informed consent, answered a questionnaire about demographic and reproductive factors (including age at menarche), and completed duplicate height and weight measurements (triplicate, if two measures were outside of acceptable repeatability). Body mass index (BMI) was calculated from measured height and weight. For girls, BMI Z-scores were calculated according to CDC reference data [18]. Tanner breast and pubic hair stages were assessed by visual inspection in all girls by the same trained research staff. Mothers and daughters also reported the percentages of all ethnic backgrounds that applied to their parents and one primary ethnicity, with which they most identified.

### DXA data collection

Although radiation exposure from DXA is extremely low (30 μSv for a full body scan and 15 μSv for one breast scan), the equivalent to the exposure from one airplane ride, a urine screening test excluded pregnancy in all participants. The recommendation for the general public is not to receive controllable radiation of more than 1,000 μSv per year. We performed DXA scans of the whole body and of both breasts (the latter used the research scan protocol). The software version 10.1 was used on a GE Lunar Prodigy Bone Densitometer (GE Healthcare). Prodigy utilizes an ultra low radiation and cadmium-zinc-telluride detector to convert X-rays into an electronic signal without the intermediate conversion to light. After changing into a hospital gown, both breasts were scanned in the decubitus mediolateral position with the nipple positioned in a true lateral profile. A duplicate breast scan of the left breast was performed on a random 10% of subjects for quality control.

**Figure 2:**
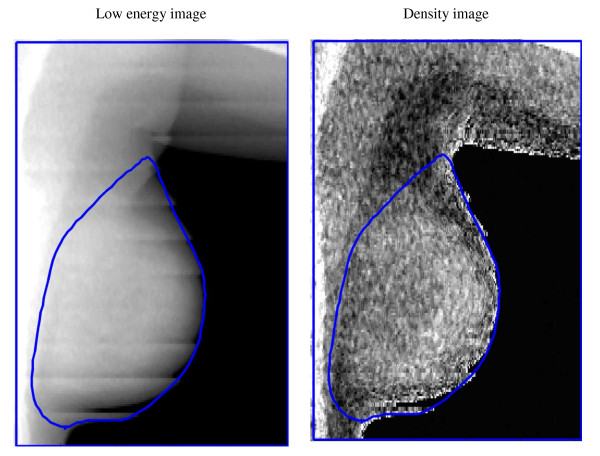
**DXA breast images: Left breast of a girl (14 years, Tanner stage 5)**.

A low-energy and a high-energy attenuation image were saved for each scan using the research mode available from GE Lunar and analyzed at the University of California at San Francisco using a Breast Density Workstation (Figure 2). The total projected breast area was manually delineated on each image by the same operator. The scans were calibrated to a 2-compartment model of fat (stearic acid) and fibroglandular tissue using custom phantoms of a known range of breast composition and thickness [14,19]. After scanning the phantoms, the "ratio value" (R-value) was defined as the ratio of the low to high energy attenuation for density and thickness. The paired R-value and high-energy attenuation value are unique for each density and thickness. Two polynomial functions are defined to convert the R-values and high-energy attenuations to either percent of fibroglandular volume (FGV) or breast thickness for each pixel. The dense breast volume is the product of the %FGV and the calculated volume of tissue in the pixel (thickness × pixel area). Given the high correlation between measures of the right and left breast, we computed mean values for total breast area, breast volume, FGV, and %FGV.

For quality control, the calibrated phantom was scanned over 8 months, once every day that participants were scheduled and once a week if nobody was scheduled. The phantom varied in thickness (2, 10, and 20 cm) and contained three %FGV values of 28%, 65%, and 100%. The precision values, i.e., test retest standard deviations, ranged from 1.9% (10 cm in thickness and 65% composition) to 5.4% (2 cm in thickness and 65% composition). For the repeated breast scans, a root mean square standard deviation of 2.5 and a correlation of 0.975 were achieved.

In addition, whole body scans were performed to determine body composition, lean soft tissue mass (kg) and fat mass (kg) of standard body regions including arms, legs, trunk, and total body. The percentage of total body fat was calculated as total fat mass (kg) divided by total lean soft tissue mass (kg) and total fat mass (kg).

### Statistical Analysis

All statistical analyses were performed using the SAS software package version 9.2 (SAS Institute, Inc., Cary, NC). To evaluate the association of breast measures between mothers and daughters, we calculated Pearson correlation coefficients and performed multiple linear regression analyses using PROC GLMSELECT to include covariates. Due to non-normal distributions, breast volume and absolute FGV were log-transformed, and %FGV was square-root transformed. The regression results are presented as standardized regression coefficients (ß), which allow the comparison of effect sizes for variables with different unit sizes because they are measured in standard deviations. The breast measures of the girls were modeled as the dependent variable, while Tanner breast stage, ethnicity, age, % total body fat, as well as the characteristics of the mothers, were treated as independent variables. Due to small numbers, Tanner breast stages were grouped into 1&2, 3, and 4&5. Since we had shown previously that parity and menopausal hormone use were not related to DXA breast density, these variables were not included [17]. Because of the small sample size, ethnicity was classified as three binary categories: all or part Caucasian, all or part Asian (Japanese, Chinese, Filipino, Korean, Other Asian), and all or part Other (Hawaiian and other Pacific Islanders, Black, Native American, Hispanic, Other). For example, if a participant reported Caucasian and Other, she was assigned "1" for Caucasian and Other and "0" for Asian. Because 12 mothers had 2 participating daughters, we repeated the models with only one of the two daughters at a time to remove the correlation associated with duplicate maternal information, but the results did not differ substantially.

## Results

The respective mean ages of mothers and daughters (Table 1) were 47.7 (range: 38.7-64.3) and 13.9 years (range: 10.2-16.9). The mean % total body fat was 39.7 ± 7.8 (range: 18.4-55.9) for mothers and 30.1 ± 9.1 (range: 13.1-54.3) for girls. The distribution of Tanner breast stages was as follows: 1 girl in stage 1, 16 in stage 2, 38 in stage 3, 13 in stage 4, and 45 in stage 5; 80 (71%) girls had reached menarche. Based on self-declared primary ethnicity, 41% of subjects were Asian, 31% Caucasian, and 28% of Other ancestry, but 38% of the mothers reported more than one ethnicity and 64% of the girls reported at least two ethnic backgrounds. Among girls, Tanner stage (*p *= 0.76) and % total body fat (*p *= 0.52) did not differ by ethnicity. The BMI Z-score was higher for girls of Other ethnicity than for girls of Caucasian and Asian ancestry (*p *= 0.04). In contrast, BMI of mothers did not differ by ethnicity (*p *= 0.14), but % total body fat was significantly higher for mothers of Other ethnicity than for mothers of Caucasian and Asian ancestry (*p *= 0.02).

**Table 1:** Characteristics of mothers and daughters

		Mothers	Daughters
N		101	113
Age (years)		47.7 ± 4.8	13.9 ± 1.7
Weight (kg)		70.8 ± 17.0	54.1 ± 14.3
Height (cm)		160.3 ± 7.3	157.4 ± 8.4
BMI (kg/m^2^)		27.5 ± 6.0	NA
BMI Z-score		NA	0.41 ± 1.02
% total body fat^b^		39.7 ± 7.8	30.1 ± 9.1
Age at menarche (years)^c^		12.5 ± 1.6	11.7 ± 1.1
Number of children		2.5 ± 1.0	---
Ethnicity, primary (N)	Caucasian	32	35
	Asian	46	41
	Other	23	37
Ethnic backgrounds reported, all or part^a^	Caucasian	49	77
	Asian	64	83
	Other	33	53
Tanner stage for breast development (N)	1-2		17
	3		38
	4-5		58

^a^All reported ethnic backgrounds; therefore, total exceeds the number of subjects.

^b^N = 100 for mothers with DXA % total body fat.

^c^N = 80 who had reached menarche.

**Figure 3:**
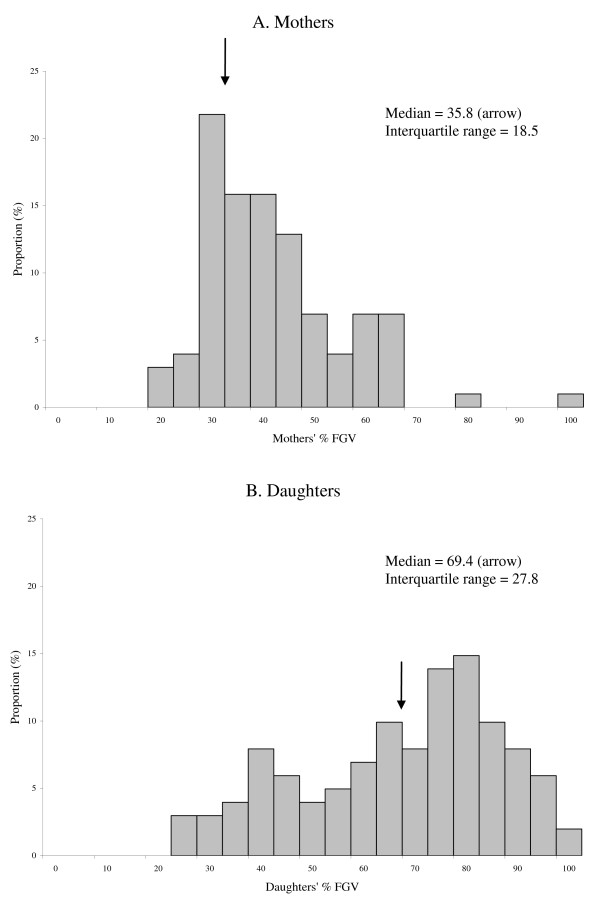
**Distributions of DXA percent breast density (%FGV) in mothers (A) and daughters (B)**.

**Figure 4:**
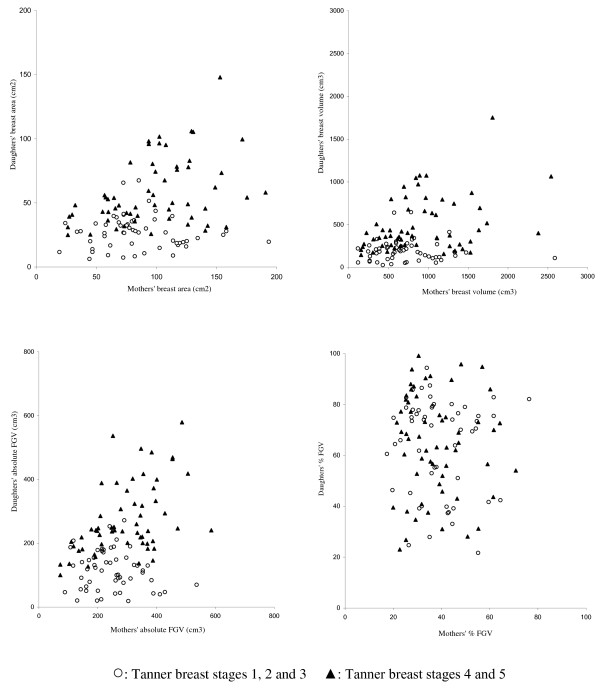
**Association of breast measures between mothers and daughters**.

As expected, total area, total volume, and absolute FGV were higher in mothers than in daughters, while %FGV was higher in daughters than in mothers (Table 2). As illustrated in Figure 3, the median and the variance, as measured by the interquartile range (IQR), were greater in daughters than in mothers. We observed statistically significant correlations between breast measures of mothers and daughters with 0.30 for breast area, 0.28 for breast volume, 0.28 for absolute FGV (*p  *< 0.01 for all), but no correlation for %FGV. Except for %FGV, the association was stronger for girls who had reached Tanner breast stages 4&5 (Figure 4); the correlations were 0.45 for breast area, 0.38 for breast volume, 0.56 for absolute FGV. In daughters, % total body fat was strongly correlated with DXA breast measures: 0.61 for breast area (*p *< 0.0001), 0.67 for breast volume (*p *< 0.0001), 0.33 for absolute FGV (*p *< 0.001) and -0.82 for %FGV (*p *< 0.0001).

**Table 2:** Breast measures for mothers and daughters

DXA measures	Mothers	Daughters	r^a^	*p *value
All mother-daughter pairs				
N	101^b^	113		
Total area (cm^2^)	92.0 ± 38.9	43.2 ± 26.4	0.30	<0.01
Total volume (cm^3^)	820.3 ± 494.1	346.7 ± 285.3	0.28	<0.01
FGV (cm^3^)	272.5 ± 107.6	196.4 ± 119.9	0.28	<0.01
%FGV	38.9 ± 14.1	64.7 ± 19.4	0.01	0.96

Mothers and daughters in Tanner stages 4&5 only				

N	54^b^	58		
Total area (cm^2^)	101.2 ± 41.4	58.7 ± 26.7	0.45	<0.001
Total volume (cm^3^)	908.5 ± 529.3	493.1 ± .317.0	0.38	<0.01
FGV (cm^3^)	297.7 ± 114.8	273.0 ± 111.5	0.56	<0.0001
%FGV	37.9 ± 12.8	64.6 ± 20.0	-0.03	0.84

^a^Pearson's correlation coefficient; total area, total volume and FGV are log-transformed and %FGV is square root-transformed in both mothers and daughters.

^b^Mothers with two daughters were included twice.

In multiple regression models of mother-daughter pairs, adjusted for ethnicity, Tanner stage, as well as % total body fat and age of mothers and daughters (Table 3), none of the three breast measures of the mothers were significantly associated with the respective measures of their daughters. The three models explained 74%, 69%, and 78% of the variance, primarily due the strong association of % total body fat and Tanner stage with the breast measures. Only when limited to the more mature girls (Tanner stages 4&5) the standardized regression estimates for breast volume (*p *= 0.02) and absolute FGV (*p *< 0.001) of the mothers became significant. However, the estimate for %FGV was not significant (*p *= 0.56). When we restricted the analysis to girls who had reached menarche (N = 80; 25 in Tanner breast stage 3 and 55 in stages 4 & 5), the results were basically the same as for the mature girls except that age of the girls was now associated with %FGV (*p *= 0.05). Analyses that included only one daughter produced similar results; substituting % total body fat with BMI Z-score or weight and height also did not improve the models.

**Table 3:** The association between breast measures of daughters and mothers^a^

		Breast volume^b^		FGV^b^		%FGV^b^	
		ß	*p *	ß	*p *	ß	*p*
All mother-daughter pairs (N = 112)^a^							
Corresponding Mother's DXA measure		0.08	0.26	0.12	0.09	-0.04	0.53
Ethnicity (all or part), daughter^c^	Caucasian	-0.06	0.26	-0.04	0.55	0.05	0.35
	Asian	0.02	0.70	0.06	0.34	0.06	0.25
	Other	0.08	0.12	0.09	0.10	0.01	0.83
DXA % total body fat, daughter		0.51	<0.0001	0.11	0.06	-0.90	<0.0001
DXA % total body fat, mother		-0.05	0.46	-0.04	0.59	0.01	0.85
Tanner breast (1&2, 3, 4&5)^d^		0.57	<0.0001	0.74	<0.0001	0.23	<0.0001
Age, daughter (years)		-0.03	0.59	0.06	0.39	0.19	<0.001
Age, mother (years)		0.10	0.05	0.11	0.05	-0.04	0.44
	*Adjusted r*^2^	0.74		0.69		0.78	

Mothers and daughters in Tanner stages 4&5 only (N = 57)^a^							
Corresponding Mother's DXA measure		0.30	0.02	0.58	<0.0001	-0.06	0.56
Ethnicity (all or part), daughter^c^	Caucasian	-0.16	0.08	-0.16	0.19	0.04	0.59
	Asian	0.03	0.74	0.08	0.49	0.04	0.55
	Other	0.10	0.26	0.17	0.14	0.01	0.96
DXA % total body fat, daughter		0.73	<0.0001	0.23	0.05	-0.90	<0.0001
DXA % total body fat, mother		-0.16	0.22	-0.15	0.27	0.06	0.53
Age, daughter (years)		-0.04	0.68	0.03	0.83	0.11	0.16
Age, mother (years)		0.14	0.16	0.25	0.06	-0.04	0.59
	*Adjusted r*^2^	0.61		0.34		0.77	

^a^Obtained by multiple linear regression with standardized regression coefficients (ß); 12 mothers with two daughters were included twice; all characteristics shown in the table are included as covariates in the model; N = 112 pairs for all mother-daughter pairs and N = 57 for mothers and daughters in Tanner stages 4&5 only due to one mother missing DXA whole body scan.

^b^In daughters, breast volume and absolute FGV were log-transformed, and %FGV was square-root transformed.

^c^Based on percentages; participants were counted more than once if they reported multiple ethnicities.

^d^Tanner breast stages were grouped into 1&2, 3 and 4&5 as a continuous varia.

## Discussion

In this comparison of DXA-based breast measures among biological mother-daughter pairs, adjusted models indicated significant associations for breast size and the amount of fibroglandular tissue in the breast, but only among the more mature girls with Tanner stages 4&5, probably because they are closer to reaching their final breast size. For %FGV, which is the ratio of fibroglandular tissue to total volume, a measure equivalent to percent mammographic density, no association was observed. As with mammographic density, body fat was inversely associated with DXA-based percent breast density in daughters (Table 3). This strong influence of adiposity as assessed by DXA % total body fat explained the correlation between mothers and daughters seen in unadjusted correlation analyses (Table 2 and Figure 4). These results suggest that the heritability of breast volume and amount of dense tissue is visible in late adolescence. However for %FGV (breast density), one may speculate that the association between mothers and daughters will only become apparent later in life when the breasts are matured as a result of pregnancies and other endogenous and exogenous influences. On the other hand, non-heritable (modifiable) factors prior to full maturation may be amenable to change during childhood.

The limited literature on the heritability of breast density did not include any girls as young as those in this report. A familial study observed a modest correlation (r = 0.27) in mammographic percent density among sisters and a non-significant association between mothers and adult daughters [9]. Our results conflict with the moderate correlation (r = 0.28) observed in an investigation of mothers and daughters that used magnetic resonance imaging (MRI) [10], though the discrepancy may be due to age differences since the daughters in the MRI study were older (20.8 ± 4.9 years) than the girls in the current study (13.9 ± 1.7 years). Their breast maturation had ended, whereas the younger girls are subject to great variability in the dense to non-dense composition of the breast until breast development reaches completion in early adulthood. Putting the two findings together, the modifiable window may extend until sometime within the 14-20 year old age range. The comparability of the MRI and DXA data is further reduced, however, by the fact that the MRI study used different compositional modeling of the tissue. While this DXA study used radiographic standards for fibroglandular and adipose tissue, the MRI study used percent water volume as a measure for fibroglandular tissue, thereby combining all water found in either fibroglandular (80%) or adipose volume (18%) into the dense compartment [14].

On the other hand, our findings agree with the distribution patterns of percent breast water as a measure of the proportion of the breast that is fibroglandular tissue in the MRI investigation, which described a larger variance in percent breast density among daughters than mothers, in particular among younger daughters (15-18 years) [10]. Our data from even younger girls support the hypothesis proposed by Boyd et al. [10] that the median density and the variance of density both decrease from adolescence to postmenopause; the median %FGV and IQR were both higher for our young girls than for the older adolescents in the MRI study. Similar to the current results, our pilot study of 13-14 year old girls also described increases in breast volume and absolute FGV by Tanner breast stage but did not observe a clear trend for %FGV [16]. Evidence from histology studies indicates that pubertal breast development involves changes in both the epithelium and the stroma and that the extension of ducts is preceded by proliferation of connective tissue including fatty tissue [20]. Given the problems of examining adolescent breast tissue, the timing of the full development of the breast gland is not well understood, but full maturation of the breast takes several years after menarche [21].

Strengths of the present study include the application of a new DXA technique that was previously tested in adolescent girls of all Tanner breast stages [16]. Although the reliability of the method varied slightly by breast thickness, the quality control results were excellent (1.9-5.4%) and we do not expect nonlinear accuracy due to thickness. Error in measuring the smaller size breasts in Tanner stages 1&2 may have led to erroneous measures of correlations. A limitation of the current study is the relatively small sample size that did not allow separate analyses by ethnicity, but the non-significant higher percent breast density among Asians agrees with previous studies among adult women [4]. The ethnic heterogeneity poses additional problems; as in genetic studies, the variations in body size associated with ethnicity may have led to confounding and population stratification [22]. The lack of information on menstrual cycle phase may have introduced some bias, but fluctuations described in the literature are fairly small [23]. As in the familial study, genetic influences on breast density may be more difficult to detect in mothers and daughters than between sisters, due to confounding by age and adiposity as well as hormonal and environmental influences and the fathers' genetic contribution [9].

## Conclusions

In this investigation, both the size of the developing breast and the amount of dense breast tissue were correlated between girls, in the later stage of pubertal development, and their mothers. The reasons for a lack of correlation in percent breast density are not clear, but may due to the young age of the girls whose growth will continue several more years or due to confounding by maternal obesity. Another potential explanation for the lack of association is the difference in parity status, a well established determinant of breast density [6], between mothers and daughters. Furthermore, ethnic heterogeneity between mothers and daughters due to paternal influences likely affected the current observations in our multiethnic population, in particular the null findings for % FGV. Since absolute breast density shows a similar association with breast cancer as percent breast density [2,3], the finding of this study that the amount of absolute fibroglandular tissue is associated between mothers and daughters supports a heritable component of breast density as shown in Caucasian populations [10-12]; as far as we know no reports for other ethnic groups have been published. In the future, such information on heritable characteristics may be useful in risk prediction models [24,25] or in efforts to modify breast cancer risk during adolescence. Future investigations using a longitudinal design and examination of siblings may be able to elucidate the changes in breast composition in this age group. The ability to examine breast tissue development during adolescence allows us to investigate risk factors, e.g., nutrition, adiposity, physical activity patterns, that influence breast cancer risk later in life [26-28].

## List of Abbreviations

BMI: Body mass index; DXA: Dual Energy X-ray Absorptiometry; FGV: Fibroglandular volume; %FGV: Percent fibroglandular volume; KP: Kaiser Permanente; MRI: Magnetic resonance imaging.

## Competing interests

The author declares that they have no competing interests.

## Authors' contributions

GM and RN conceived the research idea, obtained funding, designed the study, and oversaw the data collection. JS developed the DXA imaging method and directed the analysis of the breast scans. YM and YD coordinated the recruitment and data collection. YM performed the statistical analysis and drafted the manuscript. All authors read and approved the final manuscript.

## Pre-publication history

The pre-publication history for this paper can be accessed here:

http://www.biomedcentral.com/1471-2407/11/330/prepub
